# Systematic review and meta-analysis on physical barriers to prevent root dentin demineralization

**DOI:** 10.1038/s41598-022-22132-0

**Published:** 2022-10-28

**Authors:** R. J. Wierichs, T. Müller, G. Campus, T. S. Carvalho, S. H. Niemeyer

**Affiliations:** 1 Department of Restorative, Preventive and Pediatric Dentistry, University of Bern, zmk Bern, Freiburgstrasse 7, 3010 Bern, Switzerland; 2 Department of Surgery, Microsurgery and Medicine Sciences, School of Dentistry, University of Sassari, Viale San Pietro 3/c, 07100 Sassari, Italy; 3 Faculty of Dentistry, Sechenov First Moscow State Medical University, Moscow, Russia 119991

**Keywords:** Ageing, Gerodontics, Preventive dentistry

## Abstract

The present review systematically analyzed in vitro and in situ studies investigating physical diffusion barriers (sealants, desensitizer or adhesives) to prevent the development or the progression of root (dentin) demineralization. Three electronic databases (PubMed-Medline, CENTRAL, Ovid-EMBASE) were screened for studies from 1946 to 2022. Cross-referencing was used to identify further articles. Article selection and data abstraction were done in duplicate. Languages were not restricted. The type of outcome was not restricted, and their mean differences (MD) were calculated using fixed- or random-effects models. Risk of Bias was graded using Risk of Bias 2.0 tool. From 171 eligible studies, 34 were selected for full-text analysis evaluating 69 different materials, and 17 studies—still evaluating 36 different materials—were included (3 in situ and 14 in vitro). Ten studies evaluated desensitizers; 8 adhesives; and 1 infiltration. Meta-analyses were possible for all 17 studies. Meta-analyses revealed that lesion depth after no treatment was significantly higher than after the application of single-step adhesives (MD[_95%_CI] = − 49.82[− 69.34; − 30.30]) and multi-step adhesives (MD[_95%_CI]=–60.09 [–92.65, –27.54]). No significant differences in the lesion depth increase between single- and multi-step adhesives could be observed (MD[_95%_CI]=30.13 [–21.14, 81.39]). Furthermore, compared to no treatment the increase of the lesion depth was significantly hampered using desensitizers (MD[_95%_CI] = − 38.02[− 51.74; − 24.31]). Furthermore, the included studies presented unclear or high risk. A physical diffusion barrier can significantly hamper the increase of lesion depth under cariogenic conditions. Furthermore, multi-step adhesives seem not to be more effective than single-step adhesives. However, this conclusion is based on only few in vitro and in situ studies.

## Introduction

Life expectancy has gradually increased in many countries, bringing along many new health vulnerabilities, also regarding oral health. The elderly can present decreased motor skills^1^, resulting in difficulties to perform a proper oral hygiene, increasing the susceptibility to caries^2^. The prevalence of root caries is further propelled in this age group, since the elderly show higher indices of gingival recession and root exposure^3^, and also a reduced salivary secretion^4^. Consequently, several non-invasive approaches have been tested to prevent the development or to inactivate Root Caries Lesions (RCL)^5,6^, though not all were (completely) successful. Therefore, other micro-invasive strategies have been tested to further prevent RCL.

Dental sealants showed clinically significant results in reducing the incidence of pit and fissure caries^7^, proximal caries^8^ as well as the development of white spot lesions during orthodontic treatments with fixed appliances^9^. Sealants have also been tested on dentin in vitro^10–13^. Other micro-invasive strategies, such as the use of desensitizers^10,14–16^ or adhesives^13,17,18^, can also act as physical barriers that may prevent growth of biofilm by blocking nutrition. Nonetheless, these diffusion barriers have solely been tested in vitro or in situ, and no quantitative data synthesis (meta-analysis) focusing on the effect of various micro-invasive strategies to prevent the development and/or the progression of RCL has been published yet.

Thus, this systematic review was designed and caried out with the aim to critically summarize and evaluate results of in vitro and in situ studies investigating physical diffusion barriers (*e.g*. sealants, desensitizers or adhesives) to prevent the development or the progression of root (dentin) demineralization.

## Methods

### Review design

This review aimed at systematically retrieving and analyzing in vitro and in situ studies assessing physical diffusion barriers (e.g. sealants, desensitizer or adhesives) to reduce or arrest the development or the progression of root (dentin) demineralization. The review was conducted according to the guidelines by the Cochrane Collaboration^19^; reporting followed the PRISMA statement (Preferred Reporting Items for Systematic Reviews and Meta-analyses) (please see Supplementary Material)^20^. Since this is an review on in vitro and in situ studies and since no study registration is necessary for this type of review it was not registered in e.g. prospero.

### Inclusion and exclusion criteria

Based on the following PICOS (Participants, Intervention, Outcome, Study design) schema, in vitro and in situ studies assessing the effect of any kind of physical diffusion barrier on root (dentin) demineralization were included (Table 1).

**Table 1:** PICOS schema: Population (P), Intervention (I), Comparison (C), Outcomes (O) and Study Design (S).

**P**	‘Participants’: dentin specimens undergoing a cariogenic challenge
**I**	Intervention: application of any kind of physical diffusion barrier (e.g. sealants, desensitizers or adhesives)
**C**	Control: specimens undergoing a cariogenic challenge not being protected with a physical diffusion barrier (untreated control) or specimens with another kind of diffusion barrier (e.g. adhesive vs. desensitizer)
**O**	Outcome: development (development and progression) of dentin caries lesions assessed by radiography (transversal microradiography, micro-computed tomography), scanning electron microscope (SEM; e.g. lesion depth, dentinal tubule occlusion) or (inverse) polarizing microscopy
**S**	Studies: (non-)randomized controlled in vitro and in situ studies

The following inclusion criteria were adopted:

- Controlled in vitro and in situ studies on dentin specimens undergoing a cariogenic challenge (no further specification regarding e.g. minimum follow-up period, minimum number of specimens, etc. were made)
- assessment of different physical diffusion barriers (e.g. sealants, desensitizers or adhesives)
- assessment of root (dentin) demineralization (development and/or regression)
  The following exclusion criteria were adopted:
- outcomes not assessing root (dentin) caries
- ‘single group studies’/studies without any control group

### Literature sources

Two authors (TM, RJW) independently reviewed the title and abstract of articles retrieved following a defined search strategy (Supplementary Table 1). The reviewers were not blinded to journal names nor to article authors. No limitations concerning language or status were applied. Grey literature was not evaluated. The electronic search was conducted through PubMed-Medline, CENTRAL, Ovid-EMBASE for studies from 1946 to August 29th 2022 and the results of searches were cross-checked to eliminate duplicates. A detailed sequence of filtering search results to include relevant articles can be found in the supplementary document.

At first the titles and abstracts of the searched articles were examined independently by two authors (TM, RJW). Any disagreements in the eligibility criteria were solved by discussion and if no consensus was reached, a third author (SHN) was consulted. Then, selected studies were screened full-text. Cross-referencing was performed to identify further relevant articles that could fulfil the inclusion criteria.

### Data extraction

Two authors (TM, RJW) extracted the data by means of predefined structured tables (Microsoft Excel, Microsoft Corporation, Redmond, USA)^21,22^. For each study, the following data were systematically extracted:

- study type and setting
- treatment and control groups
- type of intervention: physical diffusion barrier (sealants, desensitizer, adhesive, etc.)
- product brands
- follow-up time/study duration
- primary and secondary outcomes
  - lesion depth
  - mineral loss
  - dentinal tubule occlusion
  - antibacterial activity
  - etc.
- number of participants and specimens being included
- type of teeth used (bovine vs. human)
- type of baseline condition (sound dentine or pre-demineralized dentin)
- sample size
- numeric and narrative main results

### Risk of bias assessment

Two authors (TM and RJW) independently evaluated the risk of bias. Any disagreement between the reviewers was discussed until an agreement was reached and if needed, by consulting a third author (SHN). For risk of bias assessment, the guidelines by the Cochrane Collaboration^19^ were slightly adapted: risk of bias criteria being used in recent systematic reviews of in vitro studies were added^23,24^. Thus, risk of bias assessment included:

- random sequence generation
- allocation concealment
- blinding of participants and personnel
- blinding of outcome assessment
- incomplete outcome data
- selective outcome
- description of sample size calculation
- use of teeth with similar dimensions
- use of caries lesions (artificial or natural) with similar dimensions
- treatment performed by the same operator
- materials used according to the manufacturers’ instructions
- Anything else ideally prespecified (conflict of interest, sponsored by manufacturer)

### Data analysis

The statistical analyses were conducted in Review Manager (RevMan version 5.4 software, Cochrane Collaboration, Copenhagen, Denmark, 2014)^25^. Fixed or random-effects meta-analyses were performed depending on heterogeneity (I^2^ < 35%: fixed-effects; I^2^ > 35%: random-effect)^9,26^. Statistical significance was defined as *p* value ≤ 0.05 (Z test) and heterogeneity was assessed with I^2^. Forest plots were created to illustrate the meta-analysis.

For continuous variables, the primary measures of effect between treatment and control groups were the mean differences (MD) for studies using the same outcome and standardized mean differences (SMD) for studies using the same construct but different scales^21,27^.

### Assessment of reporting bias

In the presence of more than 10 studies in a meta-analysis, the possible presence of publication bias was investigated for the primary outcome. Publication bias was assessed by Funnel plots^28^.

### Sensitivity analysis

We explored whether or not the analysis of studies stratified by (1) risk of bias yielded similar or different results. For this studies at high risk of bias were eliminated in a second/third analysis.

### Statement of ethics

This article does not contain any studies with human participants or animals performed by any of the authors.

## Results

**Figure 1:**
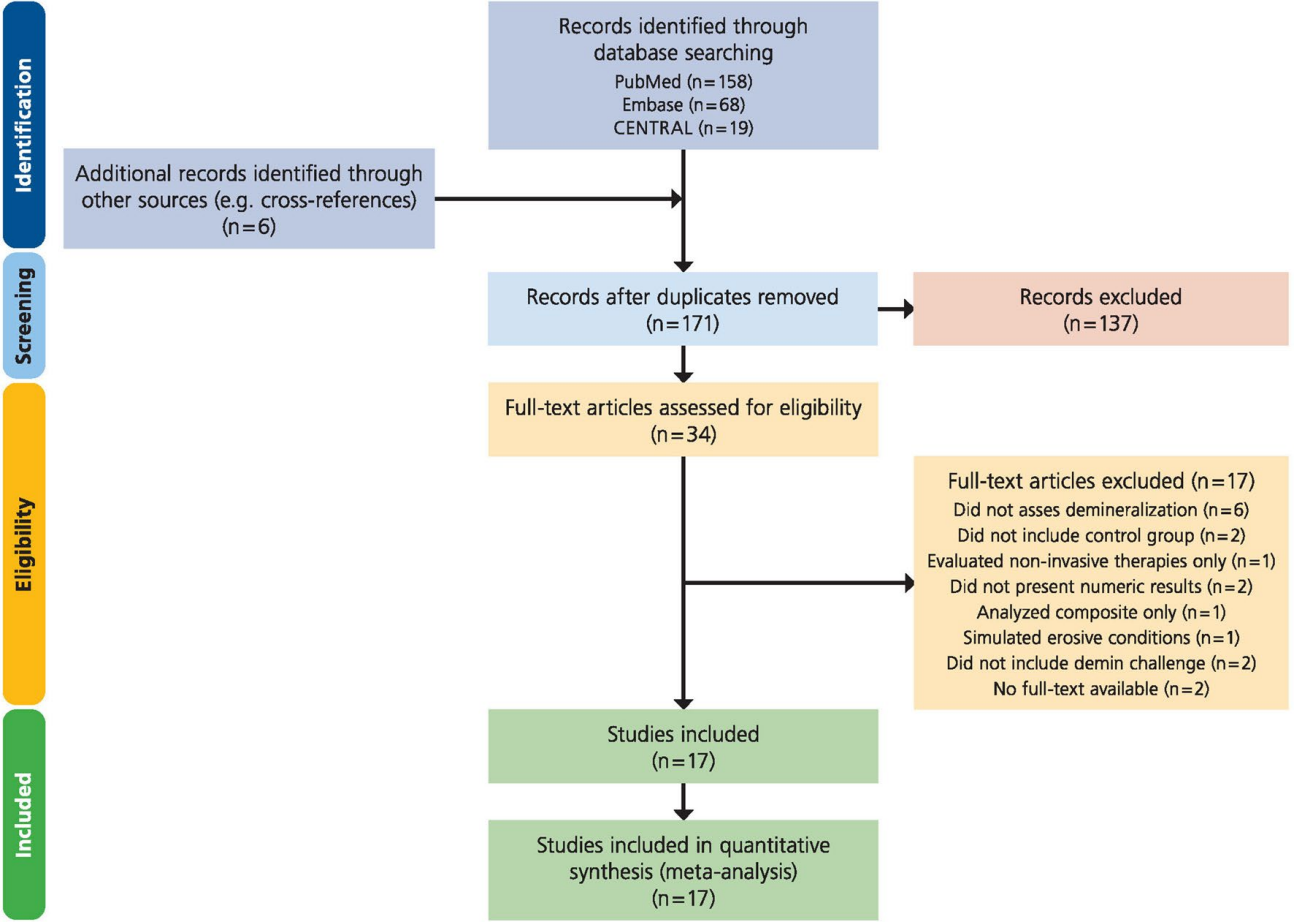
PRISMA flow diagram.

A total of 171 studies were initially identified, and after title and abstract screening, 34 studies analyzing 69 different materials were assessed for eligibility (Fig. 1). After full-text screening 17 studies had to be excluded (Supplementary Table 2) and 17 studies—still evaluating 36 different materials—were included^10,11,14,15,29–41^. Characteristics of the included studies are shown in Table 2. Three studies were in situ studies^14,15,29^ and 14 were in vitro studies.

**Table 2:** Detailed summary of included studies.

Study	In vitro/in situ	Type of intervention	Independent control/within sample control	Material groups	One step/multiple step (with etch)	Results	Sealing of sound surface (s)/sealing of artificial lesion (l)
Bekes, Francke et al., 2009	In situ	1) Control 2) Hyposen	Independent	1) – 2) Desensitizer	1) No treatment 2) Multiple step (2 steps, 2layers)	The application of a desensitizer hampers the demineralization of dentin	s
Bekes, Schmelz et al., 2009	In situ	1) A: Admira Protect 2) V: VivaSens 3) H: Hyposen 4) C: control group (untreated)	Independent	1) Desensitizer 2) Desensitizer/(Fluoride) 3) Desensitizer 4) –	1) One step (2 layers) 2) One step (Mix 2 solutions) 3) Multiple step (2 steps, 2 layers) 4) No treatment	The application of a desensitizer hampers the demineralization of dentin under different oral hygiene conditions	s
Gernhardt et al., 2004	In vitro	1) Syntac, non-irradiated 2) Syntac, irradiated 3) Scotchbond 1, non-irradiated 4) Scotchbond 1, irradiated	within sample	1) Adhesive 2) – 3) Adhesive 4) –	1) Multiple step (2 steps) 2) Multiple step (2 steps) 3) Multiple step (2 steps, 2 layers) 4) Multiple step (2 steps, 2 layers)	The lesion depth was significantly reduced compared to the control groups No significant difference between the irradiated and non-irradiated specimens	s
Gernhardt et al., 2005	In vitro	1) Seal & Protect 2.0 2) D/Sense 2 3) Gluma Desensitizer	within sample	1) Sealant 2) Desensitizer 3) Desensitizer	1) One step (2 layers) 2) Multiple step (2 solution) 3) One step (1 layer)	The application of a desensitizer hampers the demineralization of dentin	s
Gernhardt et al., 2007	In situ	1) C: control group (untreated) 2) S: Syntac Classic 3) X: Xeno III 4) H: Hyposen	Independent	1) – 2) Adhesive 3) Adhesive 4) Desensitizer	1) No treatment 2) Multiple step (2 steps) 3) One step (Mix 2 solutions) 4) Multiple step (2 steps, 2 layers)	The application of a desensitizer/adhesive hampers the demineralization of dentin	s
Hahn et al., 1999	In vitro	1) Group 1: Syntac, Heliobond (no air thinning) 2) Group 2: Syntac, Heliobond (as recommended) 3) Group 3: Syntac, without Heliobond 4) Group 4: Prime & Bond 2.0 (no air drying) 5) Group 5: Prime & Bond 2.0 (as recommended) 6) Group 6: Prime & Bond 2.0 (dentin pretreated with 36% phosphoric acid)	within sample	1) – 2) Adhesive 3) Adhesive 4) – 5) Adhesive 6) –	1) Multiple step (3 steps) 2) Multiple step (3 steps) 3) Multiple step (2 steps) 4) One step (2 layers) 5) One step (2 layers) 6) Multiple step (2 steps, 2 layers)	The application of an adhesive hampers the demineralization of dentin	s
Kawamura et al., 2019	In vitro	1) MS Coat One 2) MS Coat F 3) Fluor Jelly 4) Contol group	Independent	1) Desensitizer 2) Desensitizer 3) Desensitizer 4) –	1) One step 2) One step 3) One step 4) –	A desensitizer containing 3000 ppm fluoride and MS polymer has the same anti-demineralization effect as an fluoride paste containing 9000 ppm F	s
Kuramoto et al., 2005	In vitro	1) Prime & Bond 2.1 (PB) 2) Single Bond (SB) 3) Liner Bond 2 (LB2) 4) MDPB-containing primer and LB bond 5) Control group (untreated)	Independent	1) Adhesive 2) Adhesive 3) Adhesive 4) Adhesive 5) –	1) One step 2) One step 3) Multiple step (2 steps) 4) Multiple step (2 steps) 5) No treatment	The application of an adhesive hampers the demineralization of dentin	l
Lodha et al., 2014	In vitro	1) Control group (deionized water) 2) Duraphat (positiv control) 3) Teethmate Desensitizer 4) Nanoseal	Independent	1) – 2) Fluoride 3) Desensitizer 4) Desensitizer	1) No treatment 2) One step 3) One step 4) One step (Mix 2 solutions)	Both desensitizer and hamper the demineralization of dentin , with 4) resulting in improved inhibition after prolonged immersion in artificial saliva	s
Miyajima et al., 2016	In vitro	1) Nanoseal 2) Control group (untreated)	Independent	1) Desensitizer 2) -	1) One step (Mix 2 solutions) 2) No treatment	Calcium and phosphorous were incorporated into the superficial layer of specimens in 4)	s
Obayashi et al., 2020	In vitro	1) Control group 2) MS0 (−) 3) MS0 (+) = MS Coat One 4) MS3000 (−) 5) MS3000 (+) = MS Coat F 6) MS7000 (−) 7) MS7000 (+) 8) NaF9000 = Fluor Jelly (positive control)	Independent	1) – 2) Desensitizer 3) Desensitizer 4) Desensitizer 5) Desensitizer 6) Desensitizer 7) Desensitizer 8) Desensitizer	1) No treatment 2) One step 3) One step 4) One step 5) One step 6) One step 7) One step 8) One step	The application of an experimental polymer-based desensitizer hampers the demineralization of dentin	s
Oshima et al., 2015	In vitro	1) Control group 2) OA (1% oxalic acid ) 3) MS Coat One (MSO) 4) MS Coat F (MSF)	Independent	1) – 2) Acid 3) Desensitizer 4) Desensitizer	1) No treatment 2) One step 3) One step 4) One step	The application of a polymer-based desensitizer with sodium fluoride was effective in sealing the dentin tubules and reduce demineralization of dentin	s
Saad et al., 2019	In vitro	1) Nanoseal 2) Caredyne Shield 3) Contol group	Independent	1) Desensitizer 2) Desensitizer 3) –	1) One step (Mix 2 solutions) 2) One step (Mix 2 solutions) 3) No treatment	Application of the zinc-containing CS desensitizer may show good potential as a new therapeutic treatment to prevent root caries formation	l
Tao et al., 2020	In vitro	1) Control group (negative) 2) Scotchbond Multi-Purpose SBMP (positive control) 3) SBMP-DMAHDM 4) SBMP-NACP 5) SBMP-NACP + DMAHDM	within sample	1) – 2) Adhesive 3) Adhesive 4) Adehsive 5) Adhesive	1) No treatment 2) Multiple step (2 steps) 3) Multiple step (2 steps) 4) Multiple step (2 steps) 5) Multiple step (2 steps)	The NACP + DMAHDM adhesive was effective in remineralizing dentin lesion in a biofilm model	l
Walter et al., 2008	In vitro	1) Gluma Comfort Bond 2) Gluma Comfort Bond + Desensitizer 3) iBond 4) One-up Bond F	within sample	1) Adhesive 2) Adhesive 3) Adhesive 4) Adhesive	1) Multiple step (2 steps) 2) Multiple step (2 steps) 3) One step (3 layers) 4) One step (3 layers)	Lesions in the groups 2), 3) and 4), were shallower after treatment than in control group	s
Zhou et al., 2017	In vitro	1) Control group (untreated) 2) Clearfil SE Bond (SEB) 3) Icon-etch120s + Icon-infiltrant (HA120) 4) Icon-etch10s + Icon-infiltrant (HA10) 5) K-etchant10s + Icon-infiltrant (PA10)	Independent	1) – 2) Adhesive 3) Infiltration 4) – 5) –	1) No treatment 2) Multiple step (2 steps) 3) Multiple step (3 steps, 2 layers) 4) Multiple step (3 steps, 2 layers) 5) Multiple step (3 steps, 2 layers)	Resin infiltration with 120 s-HCl pretreatment has got a good penetration ability and preventive effect on root caries	l

Study	Duration	Outcome	Method of measurement	Number of specimen per group	Randomized allocation of specimens		
Bekes, Francke et al., 2009	35 days	Lesion depth	Polarized light microscope	9	Randomized		
Bekes, Schmelz et al., 2009	35 days	Lesion depth	Inverse polarizing microscope, Photographs	18	Randomized		
Gernhardt et al., 2004	14 days	Lesion depth	Inverse polarizing microscope, Photographs	15	Randomized		
Gernhardt et al., 2005	14 days	Lesion depth	Inverse polarizing microscope, Photographs	20	Randomized		
Gernhardt et al., 2007	35 days	Lesion depth	Inverse polarizing microscope, Photographs	28	n/a		
Hahn et al., 1999	6 days	Lesion depth	Inverse polarizing microscope	10	Randomized		
Kawamura et al., 2019	4 days	Integrated mineral loss, mineral density profile	Transverse microradiography (TMR)	6	Randomized		
Kuramoto et al., 2005	14 days	Lesion depth	Contact microradiograph, stereomicroscope, Scanning electron microscope (SEM)	5	n/a		
Lodha et al., 2014	7 days (but only 3 h demin)	Integrated mineral loss, mineral density profile surface morphology	Micro-CT, SEM	10	Randomized		
Miyajima et al., 2016	3 days	Lesion depth, Integrated mineral loss, mineral density profile	Micro-CT, SEM, Electron probe micro analyzer (EPMA)	12	Randomized		
Obayashi et al., 2020	1 day (but only 10 h demin)	Integrated mineral loss, mineral density profile, surface morphology	Micro-CT, SEM	18	n/a		
Oshima et al., 2015	1 day (but only 5 h Demin)	Integrated mineral loss, mineral density profile, surface morphology, fluoride ion release	Micro-CT, SEM, Fluoride ion-specific electrode	21	n/a		
Saad et al., 2019	0.83 day (20 h)	Lesion depth, Integrated mineral loss, surface morphology	TMR, SEM	7	Randomized		
Tao et al., 2020	3.33 days (80 h)	Lesion depth	Polarized light microscope, Photographs	10	Randomized		
Walter et al., 2008	7 days	Lesion depth, lesion expansion	Confocal laser scanning microscope (CLSM)	12–18	n/a		
Zhou et al., 2017	4 days	Lesion depth, Resin penetration, frequency of cervical enamel loss dentino-enamel-junction separation length	Swept-source optical coherence tomography (SS-OCT), Fluorescent microscope (FM), CLSM	12	Randomized		

Ten studies evaluated desensitizer^10,11,14,15,29–34^, 8 studies adhesive^14,35–41^, 1 sealants^10^ and 1 infiltrants^39^. The development of new dentin lesions was investigated in 13 studies^10,11,14,15,29–33,35,36,38,40^ whereas 4 studies^34,37,39,41^ analyzed the progression of artificial lesions. The outcomes were described by using lesion depth^10,11,14,15,29,34–41^ and mineral loss^11,30–34,41^ (Table 2).

Meta-analyses were performed for studies investigating similar interventions and outcomes in more than one study. Meta-analyses could have been performed comparing single-step adhesives versus untreated control^14,35–38,40,41^ and for multi-step adhesives versus untreated control^14,18,35–37,39,40^, single-step adhesives versus multi-step adhesives^14,35–37,40^ as well as desensitizers versus untreated control^10,11,14,15,29,34^. However, in some comparisons a few studies had to be excluded because no numeric results had been reported^42,43^, an erosive/abrasive challenge was made instead of a cariogenic one^12^ or the specimens were solely stored in remineralization solution without simulating any cariogenic challenge^18,44^.

**Figure 2:**
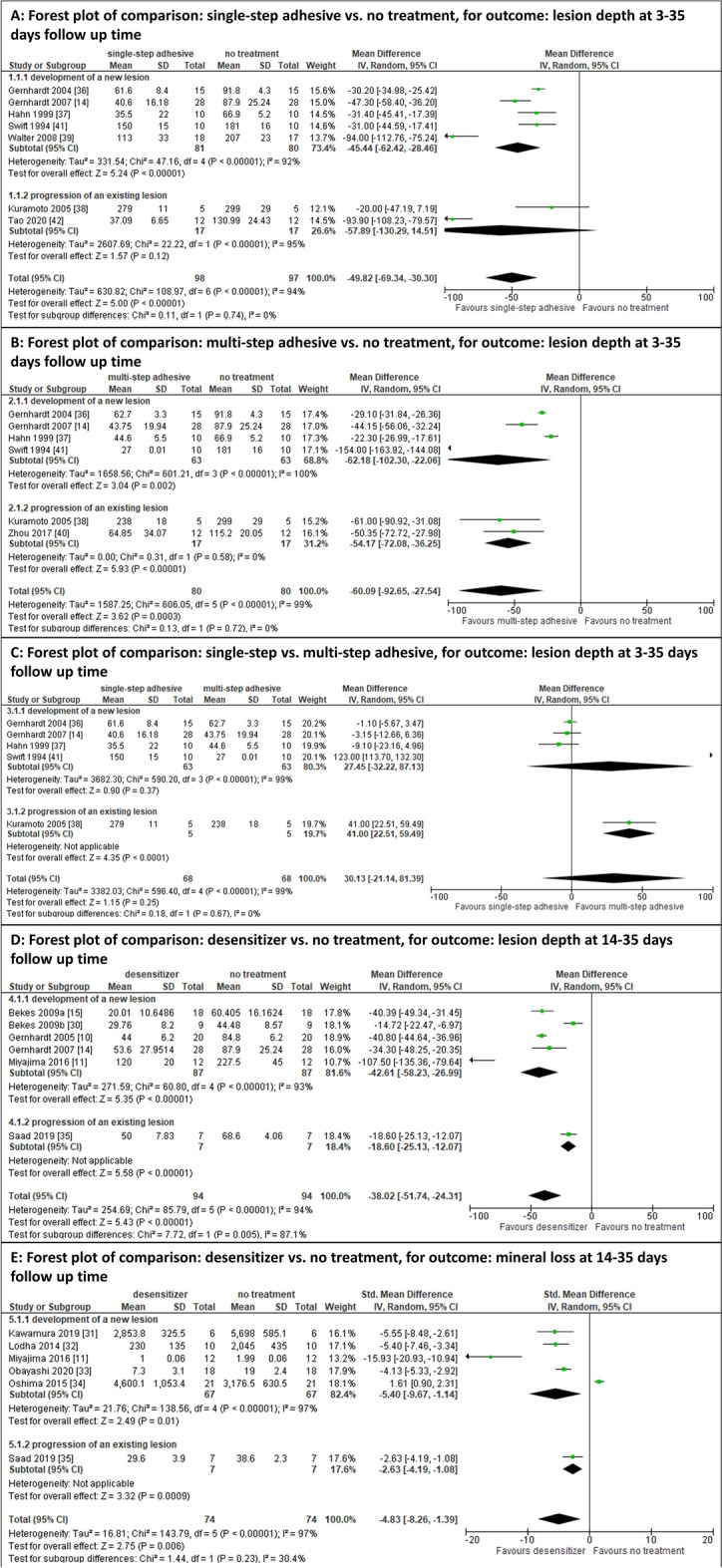
Quantitative meta-analyses for increase of the lesion depth comparing (**A**) single-step adhesives versus untreated control, (**B**) multi-step adhesives versus untreated control, (**C**) single-step versus multi-step adhesives (**D**) desensitizer versus untreated control; and (**E**) same as D, but for outcome. For each comparison MD, 95% CI, forest plots, heterogeneity parameter (I^2^) as well as overall statistics (Z, P) are shown.

From the meta-analyses, lesion depth after untreated control (no application of a physical barrier) was significantly higher than after the application of single-step adhesives (MD [_95%_CI] = − 49.82 [− 69.34; − 30.30])) and multi-step adhesives (MD[_95%_CI]=–60.09 [–92.65, –27.54]) (Fig. 2). Contrastingly, no significant differences in the increase of lesion depth between single- and multi-step adhesive was observed (MD[_95%_CI]=30.13 [–21.14, 81.39]). Furthermore, compared to no treatment, the increase of the lesion depth was significantly hampered using desensitizers (MD[_95%_CI] = − 38.02 [− 51.74; − 24.31]).

### Risk of bias analysis

**Figure 3:**
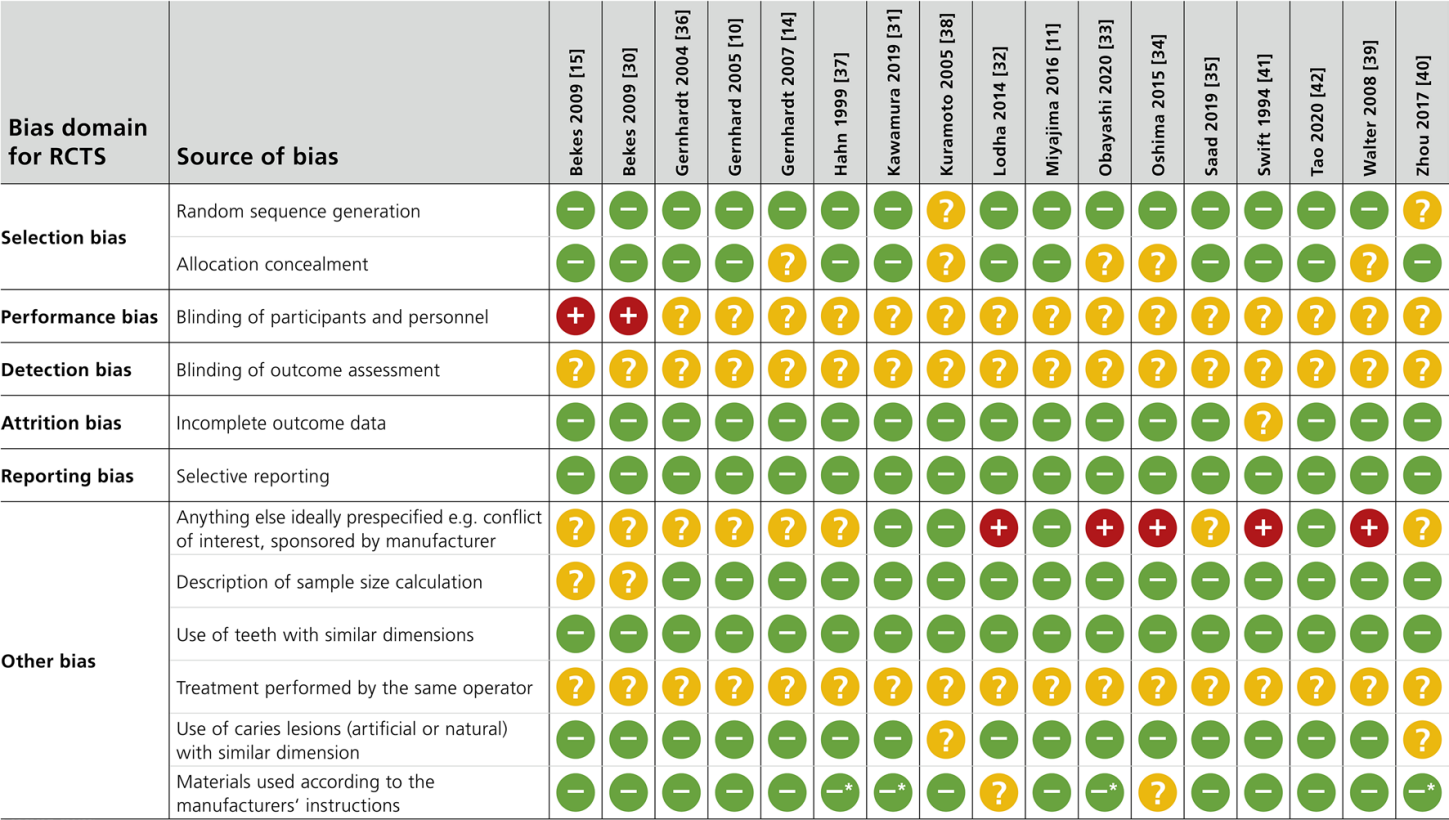
Risk of included studies. − high; + low; ? unknown. *in one group the material was used as recommended by the manufacture, in a second group the application of the tested material was modified.

Risk of bias was assessed for all 17 studies included in the meta-analysis (Fig. 3). Regarding the performance and detection bias, all studies showed an unclear risk of bias (except two studies showing a high risk of performance bias^15,29^). Furthermore, 5 studies were sponsored by the manufactures of the tested products^31–33,38,40^. Since no further information was presented about the independence of the study, the domain “other bias” was rated as high risk of bias. Overall risk of bias was low for 15 and unclear for 2 studies.

### Sensitivity analysis

When excluding studies at high risk the meta-analysis did not change.

## Discussion

The present review investigated the caries-preventive effect of different sealants, desensitizers and adhesives. A total of 17 in vitro and in situ studies were extracted from the literature, which analyzed 36 materials investigating the prevention of development or progression of root (dentin) demineralization. This reflects that no gold standard therapy has been established yet, neither on in vitro nor in situ studies. All tested materials acting as physical barriers were able to significantly hamper cariogenic lesions.

The materials significantly decreased the development of artificial root (dentin) caries lesion when compared to their non-use. This is in line with previous reviews on clinical studies analyzing sealants^7–9^, which observed that at 24 months follow-up, the use of occlusal (resin) sealants significantly reduced the incidence of fissure caries ; after a mean follow-up of 25 months a superior efficacy of proximal sealants or infiltrants over non-invasive treatments (including dietary control, biofilm control or control of de- and remineralization) was also observed; and at a median follow-up time of more than one year (12.75 months) coating agents significantly reduced the incidence of post-orthodontic white spot lesions. However, it has to be mentioned that these three reviews^7–9^ analyzed clinical studies on enamel lesions, whereas the present study solely concentrates on dentin lesions in vitro and in situ. Nonetheless, all reviews indicate that physical diffusion barriers seem to be able to prevent the development or progression of caries lesions on both enamel and dentin, by blocking bacteria nutrition and by impeding acid diffusion into the hard tissue, thus preventing (further) mineral loss.

Desensitizers are mainly used on exposed dentin to reduce dentin hypersensitivity. Nevertheless, in 10 studies, they were also tested as agents to protect against dentin demineralization. A total of 11 different desensitizers were evaluated, including light curing materials, with or without fluoride release, and different active compounds. Noteworthy, the present meta-analyses showed that the use of desensitizers significantly hampered the progression of caries lesions on dentin when compared to no treatment. This is probably due to the formation of a physical diffusion barrier from the active ingredients and the presence of fluoride, the influence of these variables was not verified in the present meta-analyses.

Glass ionomer cements (GIC) are commonly used to restore (sometimes invasively) root caries lesions^45,46^. Annual failure rates had an impressive range differed between 2.4 and 44% and success rates were significantly lower than for composite restorations^6^. Interestingly, in one of the included studies^18^, GIC was also used as a micro-invasive strategy to provide a physical barrier, though it was difficult to apply in thin layers and virtually impossible to create a smooth surface. Nonetheless, GIC was included as positive control because of its ability to release fluoride and adhesion to tooth structures^47^. Under bacteria-free and solely remineralization conditions, specimens treated with GIC showed the highest mineral change, indicating remineralization, and the highest fluoride uptake. However, the mechanical stability or retention of the thin GIC layer and the surface roughness/smoothness was not analyzed. Thus, it remains unclear if GIC could be applied in thin layers to successfully provide a physical barrier in vivo.

In recent years resin infiltration—which was primarily developed to arrest approximal (enamel) caries lesions^48^—has also been shown to mask white spot lesions^26^. After polymerization the infiltrant occludes diffusion pathways for cariogenic acids and dissolved minerals, thus acting as a physical barrier that, hypothetically, can also be applied to dentin lesions. However, the pores of demineralized dentin are larger than those in demineralized enamel, which offer a path for facilitated transport of dentinal fluid^49^, thus affecting the resin infiltration process. So, theoretically, no capillary forces could arise in demineralized dentin. Nonetheless, resin infiltration has been used in one of the included studies as micro-invasive strategy to provide a physical barrier^39^, but instead of acting only on the surface (like in the case of the other materials), it acts inside the lesion body. Interestingly, the resin infiltration formed inhomogeneous penetration layers in demineralized dentin, though still significantly reducing the increase of lesion depth when compared to the untreated control group. Since human demineralized dentin was infiltrated in vitro, it might be speculated that, firstly, capillary forces might arise when dentin fluid is not simulated—as it was the case in the abovementioned study, and secondly, that hybridization by resin interdiffusion into the exposed dentinal collagen layer, combined with attachment of resin tags into the opened dentin tubules, cannot only be observed after the application of dentin adhesives^50^ but also after resin infiltration.

In the present study, the Cochrane Collaboration’s tool for assessing risk of bias was specifically adjusted for in vitro and in situ studies. For this, the criteria were complemented by relevant criteria being identified in previous systematic reviews of in vitro studies^23,24^, with the assessment consisting of eleven criteria. Overall risk of bias was unclear for two studies^31,33^ and low for the other 15 studies. Nonetheless, the included studies still presented unclear or high risk for several of the domains. Lack of information about blinding of personnel, outcome assessment and any conflict of interest were the main reasons for high/moderate risks and should be carefully considered in future studies.

There are several limitations in the present meta-analysis. Firstly, the results were obtained from in vitro and in situ studies, since until now no in vivo study has investigated physical diffusion barriers to reduce or arrest the development or the progression of root (dentin) demineralization. Secondly, in most of the studies, the control group consisted of a separated group, but in few studies, the control was a protected area within each specimen. Moreover, in most of the studies the test agents were applied on sound dentin surfaces, and sometimes on artificial dentin lesions. Thirdly, the pH-cycling conditions varied between the studies. Constant demineralizing conditions were mostly used, and intermittent demineralization conditions to simulate oral pH fluctuations were used only in a few studies.Within the limitations of this systematic review, it can be concluded that physical diffusion barriers can significantly hamper the development or the progression of root (dentin) demineralization on in vitro and in situ models. Furthermore, single-step adhesives seem not to be more effective than multi-step adhesives. Nonetheless, results should be interpreted with caution, due to the low numbers of in vitro and in situ studies.

## Supplementary Information

Supplementary Information.

## Data Availability

All data generated or analyzed during this study are included in this article [and/or] its supplementary material files. Further enquiries can be directed to the corresponding author.
