# Supplementary material for: Systematic review and meta-analysis on physical barriers to prevent root dentin demineralization
Source: Sci Rep. 2022 Oct 28;12:18194. doi: 10.1038/s41598-022-22132-0 (PMC9616813; doi:10.1038/s41598-022-22132-0)
Supplement: Supplementary file 1 — Supplementary Information. [file 41598_2022_22132_MOESM1_ESM.docx]

**Systematic review and meta-analysis on physical barriers to prevent root dentin demineralization**

**R. J. Wierichs^1^, T. Müller^1,*^, G. Campus^1,2,3^, T. S. Carvalho^1^, S. H. Niemeyer^1^**

^1^ Department of Restorative, Preventive and Pediatric Dentistry, zmk bern, University of Bern, Switzerland

^2^ Department of Surgery, Microsurgery and Medicine Sciences, School of Dentistry, University of Sassari, Viale San Pietro 3/c, 07100 Sassari, Italy

^3^ Faculty of Dentistry, Sechenov First Moscow State Medical University, Moscow 119991, Russia

**Short title:** anticaries effect of dentin sealants, desensitizers and adhesives

**Keywords:** dentin; demineralization; tooth sealant; adhesives; review; meta-analysis; in vitro

***Correspondence:**

PD Dr. Richard J. Wierichs

Department of Restorative, Preventive and Pediatric Dentistry,

University of Bern, zmk Bern,

Freiburgstrasse 7,

3010 Bern, Switzerland

Tel.: +41 31 632 25 80

Fax: +41 (0) 31 632 98 75

E-mail: Richard.Wierichs@zmk.unibe.ch

**Supplementary material**

Sequence of filtering search results

The sequence of filtering search results in order to include relevant articles in the review was as follows:

1. articles written in German, French, Portuguese, Italian or English,
2. articles written in languages of which the authors were able to get a vague idea of the content (Latin-based languages) or those having only a translated (English, French, Portuguese, Italian or German) abstract,
3. articles written in languages the authors were not able to understand at all.

After deciding on the German, French, Portuguese, Italian and English articles the authors read through the category 2) articles realizing that none of them fulfilled inclusion criteria. Of course, for all category 3) articles, the authors were not able to make a decision based on their content.

**Supplementary table 1:** Search strategy as used for Pubmed

| Search | Query | Results |
| --- | --- | --- |
| #1 | root caries | 3,901 |
| #2 | dentin tubules | 3,254 |
| #3 | dentin | 41,878 |
| #4 | ((root caries) OR (dentin tubules)) OR (dentin) | 44,896 |
| #5 | adhesive | 379,63 |
| #6 | desensitizer OR desensitiser | 41,314 |
| #7 | infiltrant | 449 |
| #8 | infiltration | 258,882 |
| #9 | coating | 188,748 |
| #10 | ((((adhesive) OR (desensitizer)) OR (infiltrant)) OR (infiltration)) OR (coating) | 831,393 |
| #11 | in vitro | 1,829,827 |
| #12 | in situ | 384,333 |
| #13 | (in vitro) OR (in situ) | 2,176,519 |
| #14 | demin* | 927,892 |
| #15 | lesion | 52,414 |
| #16 | remin* | 17,863 |
| #17 | ((demin*) OR (lesion)) OR (remin*) | 990,416 |
| #18 | ((((((root caries) OR (dentin tubules)) OR (dentin)) AND (((((adhesive) OR (desensitizer)) OR (infiltrant)) OR (infiltration)) OR (coating)))) AND ((in vitro) OR (in situ))) AND (((demin) OR (lesion)) OR (remin)) | 454 |

**Supplementary table 2**: Excluded studies

| Author (Year) | Reason for exclusion |
| --- | --- |
| Endo et al., 2013^S1^ | Did not asses demineralization |
| Fu et al. 2007 ^S2^ | Did not asses demineralization |
| Grogono et al., 1994 ^S3^ | Did not present numeric results |
| Han et al., 2013 ^S4^ | No full-text available |
| Han et al., 2015 ^S5^ | No full-text available |
| Jung et al., 2019 ^S6^ | Did not asses demineralization |
| Kolker et al., 2002 ^S7^ | Did not asses demineralization |
| Kwong et al., 2000 ^S8^ | Did not include demin challenge |
| Moussa et al., 2019 ^S9^ | Analyzed composite only |
| Ogihara et al., 2021 | Did not asses demineralization |
| Okuyama et al., 2016 ^S10^ | Did not include demin challenge |
| Prabhakar et al., 2014 ^S11^ | Did not present numeric results |
| Schupbach et al., 1997^S12^ | Did not include control group |
| Wegehaupt et al., 2017 ^S13^ | Did not asses demineralization |
| Zhang et al., 2015 ^S14^ | Did not include control group |
| Zhao et al., 2020 ^S15^ | Simulated erosive conditions |
| Zhao et al., 2016 ^S16^ | Evaluated non-invasive therapies only |

**References**

S1 Endo, H. *et al.* Evaluation of a calcium phosphate desensitizer using an ultrasonic device. *Dent Mater J* **32**, 456-461, doi:10.4012/dmj.2012-308 (2013).

S2 Fu, B., Shen, Y., Wang, H. & Hannig, M. Sealing ability of dentin adhesives/desensitizer. *Oper Dent* **32**, 496-503, doi:10.2341/06-143 (2007).

S3 Grogono, A. L. & Mayo, J. A. Prevention of root caries with dentin adhesives. *Am J Dent* **7**, 89-90 (1994).

S4 Han, L. & Okiji, T. Effects of a novel fluoride-containing aluminocalciumsilicate-based tooth coating material (Nanoseal) on enamel and dentin. *Am J Dent* **26**, 191-195 (2013).

S5 Han, L. & Okiji, T. Dentin tubule occluding ability of dentin desensitizers. *Am J Dent* **28**, 90-94 (2015).

S6 Jung, J. H. *et al.* Dentin sealing and antibacterial effects of silver-doped bioactive glass/mesoporous silica nanocomposite: an in vitro study. *Clin Oral Investig* **23**, 253-266, doi:10.1007/s00784-018-2432-z (2019).

S7 Kolker, J. L., Vargas, M. A., Armstrong, S. R. & Dawson, D. V. Effect of desensitizing agents on dentin permeability and dentin tubule occlusion. *J Adhes Dent* **4**, 211-221 (2002).

S8 Kwong, S. M., Tay, F. R., Yip, H. K., Kei, L. H. & Pashley, D. H. An ultrastructural study of the application of dentine adhesives to acid-conditioned sclerotic dentine. *Journal of dentistry* **28**, 515-528, doi:10.1016/s0300-5712(00)00032-4 (2000).

S9 Moussa, D. G., Fok, A. & Aparicio, C. Hydrophobic and antimicrobial dentin: A peptide-based 2-tier protective system for dental resin composite restorations. *Acta Biomater* **88**, 251-265, doi:10.1016/j.actbio.2019.02.007 (2019).

S10 Okuyama, K. *et al.* Efficacy of a new filler-containing root coating material for dentin remineralization. *Am J Dent* **29**, 213-218 (2016).

S11 Prabhakar, A. R., Dhanraj, K. & Sugandhan, S. Comparative evaluation in vitro of caries inhibition potential and microtensile bond strength of two fluoride releasing adhesive systems. *Eur Arch Paediatr Dent* **15**, 385-391, doi:10.1007/s40368-014-0127-y (2014).

S12 Schüpbach, P., Lutz, F. & Finger, W. J. Closing of dentinal tubules by Gluma desensitizer. *Eur J Oral Sci* **105**, 414-421, doi:10.1111/j.1600-0722.1997.tb02138.x (1997).

S13 Wegehaupt, F. J., Kummer, G. & Attin, T. Prevention of erosions by a surface sealant and adhesives under abrasive conditions. *Swiss Dent J* **127**, 740-747 (2017).

S14 Zhang, N. *et al.* Development of a multifunctional adhesive system for prevention of root caries and secondary caries. *Dent Mater* **31**, 1119-1131, doi:10.1016/j.dental.2015.06.010 (2015).

S15 Zhao, X., Pan, J., Malmstrom, H. S. & Ren, Y. F. Protective effects of resin sealant and flowable composite coatings against erosive and abrasive wear of dental hard tissues. *Journal of dentistry* **49**, 68-74, doi:10.1016/j.jdent.2016.01.013 (2016).

S16 Zhao, I. S. *et al.* Remineralising Dentine Caries Using Sodium Fluoride with Silver Nanoparticles: An In Vitro Study. *Int J Nanomedicine* **15**, 2829-2839, doi:10.2147/ijn.S247550 (2020).
